# Correction: Monocyte subset redistribution from blood to kidneys in patients with Puumala virus caused hemorrhagic fever with renal syndrome

**DOI:** 10.1371/journal.ppat.1009876

**Published:** 2021-08-25

**Authors:** Sindhu Vangeti, Tomas Strandin, Sang Liu, Johanna Tauriainen, Anne Räisänen-Sokolowski, Luz E. Cabrera, Antti Hassinen, Satu Mäkelä, Jukka Mustonen, Antti Vaheri, Olli Vapalahti, Jonas Klingström, Anna Smed-Sörensen

An initial is missing in the sixth author’s name. The correct name is: Luz E Cabrera. The correct citation is: Vangeti S, Strandin T, Liu S, Tauriainen J, Räisänen-Sokolowski A, Cabrera LE, et al. (2021) Monocyte subset redistribution from blood to kidneys in patients with Puumala virus caused hemorrhagic fever with renal syndrome. PLoS Pathog 17(3): e1009400. https://doi.org/10.1371/journal.ppat.1009400
