# Supplementary figures and images for: Monocyte subset redistribution from blood to kidneys in patients with Puumala virus caused hemorrhagic fever with renal syndrome
Source: PLoS Pathog. 2021 Mar 10;17(3):e1009400. doi: 10.1371/journal.ppat.1009400 (PMC7984619; doi:10.1371/journal.ppat.1009400)

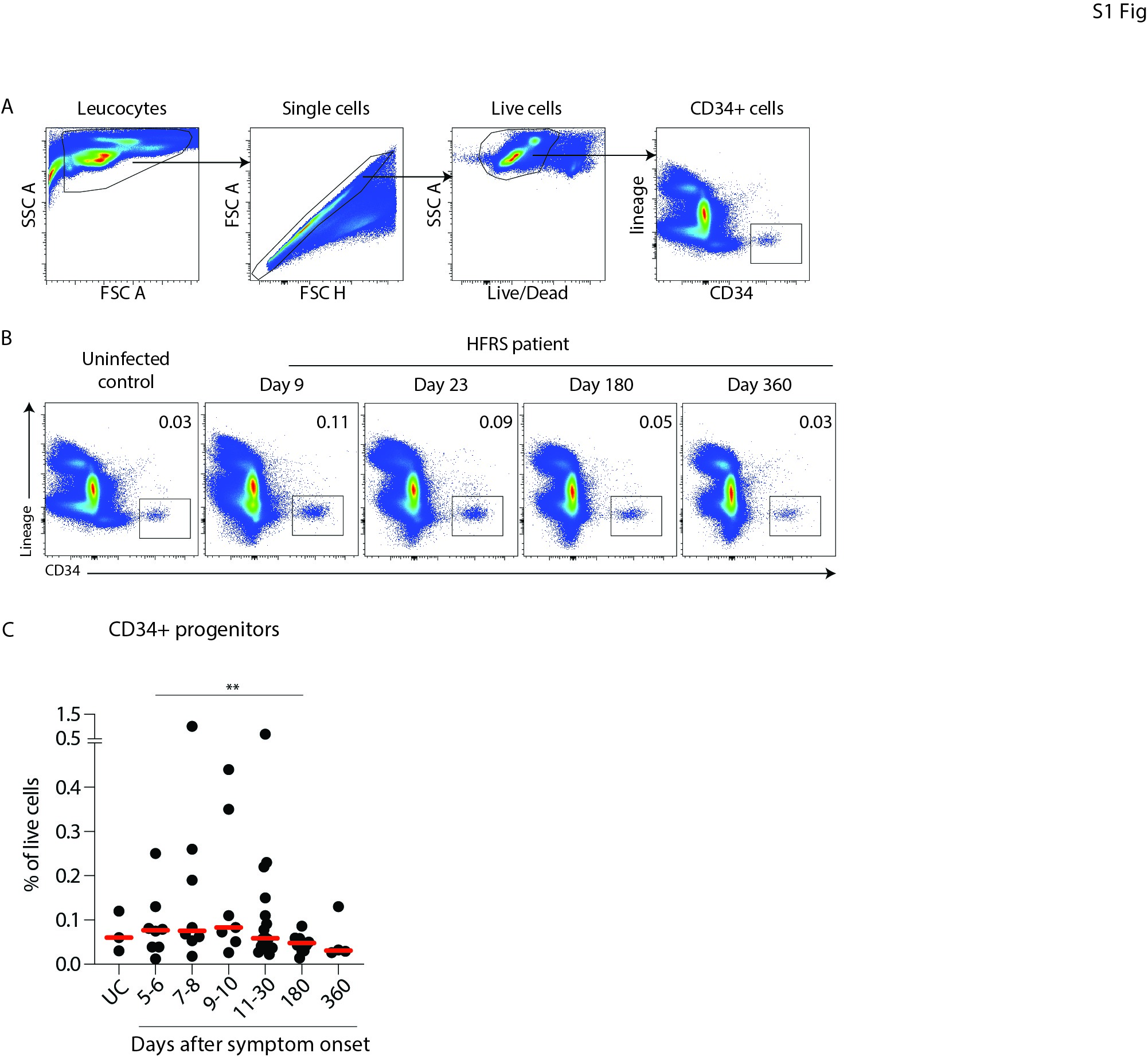

Supplement: S1 Fig — (A) Gating strategy for identification of CD34+ progenitors from PBMCs by flow cytometry. (B) Flow plots depict CD34+ progenitors in a representative uninfected control and an HFRS patient over time (day 9-day 360). (C) Graph summarizes the frequencies of CD34+ cells in UCs (n = 3) and HFRS patients (n = 16). Statistical differences between day 180 (too few data points at day 360) and other time points were assessed using a generalized estimated equation (GEE) model in SPSS and differences were considered significant at p<0.05 (**p<0.01 and ****p<0.0001). (TIF) [file ppat.1009400.s001.tif]

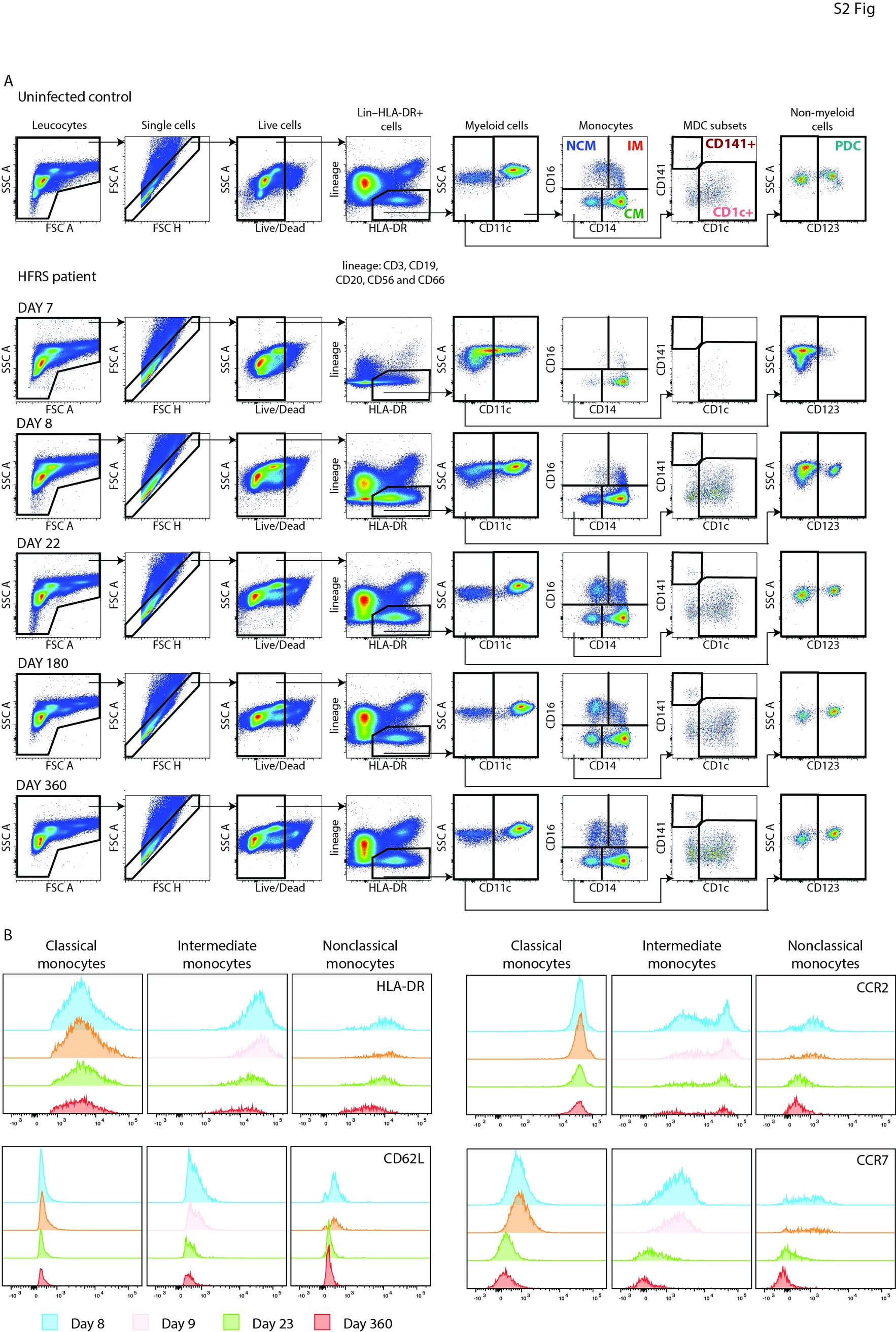

Supplement: S2 Fig — (A) From total cells, live cells were identified as single cells negative for the live/dead marker. The lineage negative, HLA-DR+ cells were identified as cells expressing HLA-DR, but not CD3, CD19, CD20, CD56 or CD66. Myeloid cells were identified as CD11c-expressing cells and from the CD11c– population, CD123+ cells were identified as plasmacytoid dendritic cells (PDCs). From the CD11c+ cells, monocyte subsets were identified as CD14+CD16– classical monocytes (CM), CD14+CD16+ intermediate monocytes (IM) and CD14–CD16+ nonclassical monocytes (NCM). Myeloid DC subsets (CD1c+ and CD141+ MDCs) were identified from the CD14–CD16– cells. (B) Histograms show the relative expression of HLA-DR, CCR2, CD62L and CCR7 in classical, intermediate and nonclassical monocytes of a single representative HFRS patient over time (day 8 to day 360). (TIF) [file ppat.1009400.s002.tif]

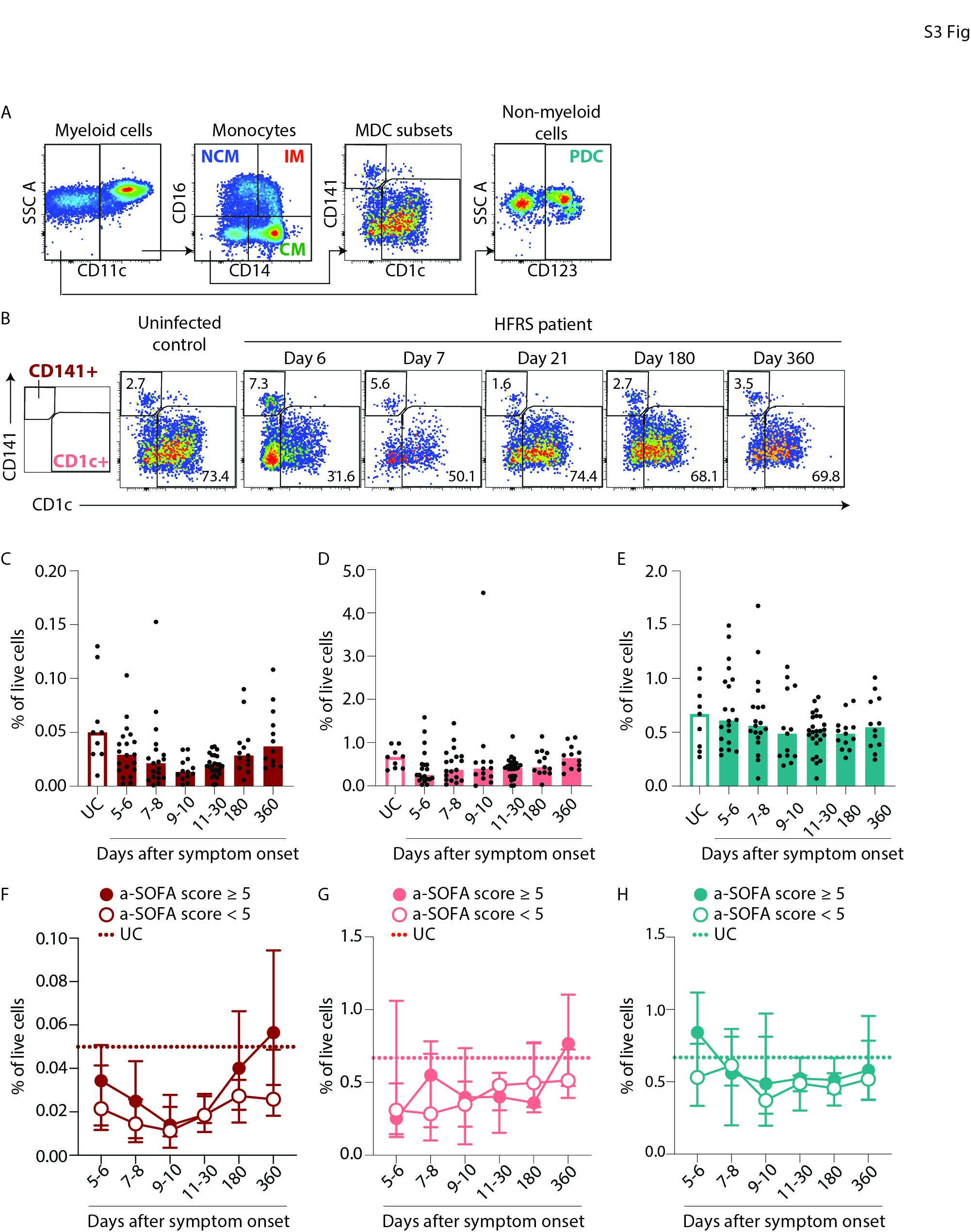

Supplement: S3 Fig — (A) Gating strategy for identification of dendritic cells (DCs) from PBMCs by flow cytometry. Representative uninfected control (UC) sample showing gating on CD11c+ myeloid cells which were negative for CD14 and CD16 to identify CD1c+ (coral) and CD141+ (maroon) myeloid DC subsets. From the CD11c– cells, CD123+ plasmacytoid DCs (teal) were identified. (B) Plots depict the myeloid DC (MDC) populations in a UC and a representative HFRS patient over the course of disease (day 6–360). (C-E) Graphs show frequencies (to total live cells) of (C) CD141+ MDCs, (D) CD1c+ MDCs and (E) PDCs in PBMCs from patients (filled bars) and HCs (empty bars). Statistical differences between day 360 and other time points were assessed using a generalized estimated equation (GEE) model in SPSS and differences were considered significant at p<0.05. (F-H) Graphs show median ± IQR frequencies of (F) CD141+ MDCs, (G) CD1c+ MDCs and (H) PDCs in PBMCs from patients stratified by severity as lower a-SOFA score (<5, empty circles) or higher a-SOFA score (≥5, filled circles). Median frequencies of respective DC subset in UCs are indicated with dotted lines. (TIF) [file ppat.1009400.s003.tif]

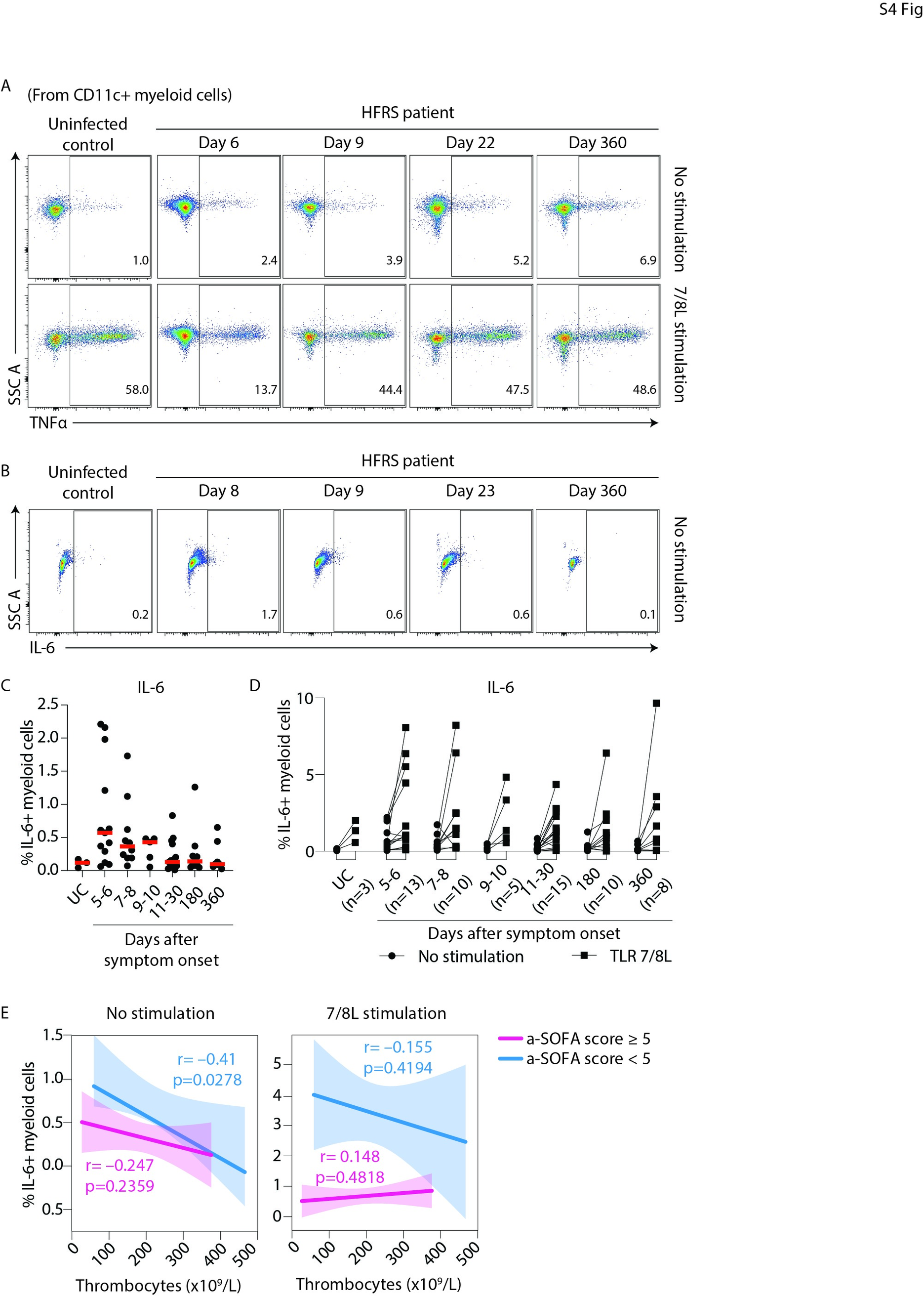

Supplement: S4 Fig — (A) Flow plots depict TNFα-producing CD11c+ myeloid cells (gated as shown in S1 Fig) in a representative uninfected control and an HFRS patient over time (day 8-day 360), in the absence (top) or presence of TLR 7/8L stimulation (bottom) in vitro for 3hr. (B) Flow plots depict IL-6-producing CD11c+ myeloid cells (gated as shown in S1 Fig) in a representative uninfected control (UC) and an HFRS patient over time (day 8-day 360), in the absence of TLR 7/8L stimulation (bottom) in vitro for 3hr, and the frequencies are summarized in (C). (D) Graph displays frequency of IL-6 producing cells in CD11c+ myeloid cells in PBMCs in UCs (n = 3) and HFRS patients (n = 15) in the absence (circle) or presence (square) of TLR 7/8L stimulation for 3 hr. Statistical differences between groups (of similar exposure conditions) were assessed by mixed-effects analysis using Dunnett’s multiple correction test and considered significant at p< 0.05. (E) Lines display bivariate linear regression analysis between thrombocyte counts (109/L) and frequency of IL-6 producing CD11c+ myeloid cell without (left) or with (right) TLR 7/8L stimulation. The shaded area represents the 95% confidence region for the fitted line. Patients are stratified by severity as lower, i.e. a-SOFA score <5 (blue) or higher, i.e. a-SOFA score ≥5 (pink). ρ represents Spearman rand statistical differences were considered significant at p<0.05. (TIF) [file ppat.1009400.s004.tif]
