# Supplementary material for: Monocyte subset redistribution from blood to kidneys in patients with Puumala virus caused hemorrhagic fever with renal syndrome
Source: PLoS Pathog. 2021 Mar 10;17(3):e1009400. doi: 10.1371/journal.ppat.1009400 (PMC7984619; doi:10.1371/journal.ppat.1009400)
Supplement: Attachment — Submitted filename: Response to reviewers_V3.docx [file ppat.1009400.s008.docx]

Saturday January 16th, 2021

Dr. Michael Malim, Editor-in-Chief

Dr. Kasturi Haldar, Editor-in-Chief

Dr. Adolfo Garcia-Sastre, Section Editor

Dr. Christina Spiropoulou, Guest Editor

PLoS Pathogens

Dear Editors,

Thank you for the constructive review of our manuscript Monocyte subset redistribution from blood to kidneys in patients with Puumala virus caused hemorrhagic fever with renal syndrome (PPATHOGENS-D-20-02196). Below, we provide a detailed point-by-point response to the specific comments of the reviewers. We have opted to merge Figures 1 and 2, thereby reducing the total number of figures to 7 but also changing the numbering of all following figures. We have clarified this to each reviewer and we hope that this does not add confusion.

In addition, we have included 3 new supplemental figures, added additional panels to 3 existing figures in the revised manuscript and 2 figures showing data for the interest of the reviewers. We have also added additional references highlighted by reviewers and discussed them in the manuscript. We have highlighted these changes in the manuscript and also described them in our reviewer responses. We believe that the revised manuscript has greatly improved by the modifications made in response to the reviewers’ comments and hope that you will consider the revised manuscript for publication in PLoS Pathogens.

**Part I - Summary
Please use this section to discuss strengths/weaknesses of study, novelty/significance, general execution and scholarship.**

**Reviewer #1:** In their manuscript entitled “Monocyte subset redistribution from blood to kidneys in patients with Puumala virus caused hemorrhagic fever with renal syndrome” Vangeti et al. analysed redistribution of monocytes in longitudinal peripheral blood samples from patients infected with Puumala virus (PUUV), which causes a mild form of hemorrhagic fever with renal syndrome (HFRS). They found that in peripheral blood of acute phase HFRS patients nonclassical monocytes (NCMs, CD14+CD16++) are depleted whereas classical (CMs, CD14++ CD16−) and intermediate monocytes (IMs, CD14++CD16+) are increased. These data are in contrast with a previous publication of the authors (Scholz et al., 2017), in which they described diminished numbers of all monocyte subsets (CM, IM, and NCM) in the peripheral blood of patients with acute HFRS. As reported previously, the expression of activation markers such as CCR7 on blood lymphocytes suggests that monocytes are activated and migrate to peripheral tissue during acute HFRS. In vitro, monocytes upregulated CCR7 after direct exposure to PUUV (CD16+ monocytes) or after contact with PUUV-infected endothelial cells (CD16- monocytes) supporting this assumption. Previously, the authors described a significant influx of monocytes expressing HLA-DR, CD11c or CD123 in bronchial tissue of patients with PUUV infection (Scholz et al., 2017). In the present manuscript, increased numbers of HLA-DR+, CD14+ and CD16+ cells were detected in the renal tissues of PUUV-infected patients. Based on their findings, the authors suggest that NCMs are recruited to the kidneys during HFRS. The analysis of kidney biopsies is interesting but incomplete. There are several problematic issues as described below.

**Author’s response:** We thank reviewer 1 for this comprehensive summary of our study. In the revised version of the manuscript, we have addressed the specific issues raised by the reviewer below.

**Reviewer #2:** The manuscript by Vangeti et al. describes experiments in which they examined the different monocyte subsets in PUUV infected patients. In the study, longitudinal peripheral blood samples and renal biopsies were examined from HFRS patients infected by PUUV and in vitro experiments were performed. This type of study can provide insights into the immunopathogenesis of hemorrhagic fever with renal syndrome caused by PUUV. The manuscript is concise and well written. In my opinion, the manuscript provide some answers of what is the role of monocytes in PUUV caused HFRS. Immunopathogenesis is an important area of research in the hantavirus field. These results are important to share and merit publication, however I have some minor comments and suggestions.

**Author’s response:** We thank reviewer 2 for this positive summary of our study.

**Reviewer #3:** The authors report declines of CD16+ monocytes in the blood during the acute phase of HFRS illness and this correlated with increased CCR2 expression in this cell population c/w the hypothesis that these cells could be getting recruited to sites of inflammation from the blood stream. Of particular interest was the inclusion of pathologic specimens from renal biopsies of HFRS patients showing increased HLADR, CD14 and CD16 as compared to other renal pathologies. The reported vitro experiments are of limited value to this study given the difficultly of interpreting their relevance to in vivo conditions. Nevertheless, this is a well written and important study of monocytes in patients with HFRS. These are difficult to perform studies given the rarity of this disease and the challenges associated with human subjects research.

**Author’s response:** We thank reviewer 3 for their positive summary of our study.

**Part II – Major** **Issues: Key Experiments Required for Acceptance**

Please use this section to detail the key new experiments or modifications of existing experiments that should be absolutely required to validate study conclusions. Generally, there should be no more than 3 such required experiments or major modifications for a "Major Revision" recommendation. If more than 3 experiments are necessary to validate the study conclusions, then you are encouraged to recommend "Reject".

**Reviewer #1:**

**Point 1:**

In Figure 2D the differences between patients a-SOFA score < 5 and patients with a-SOFA score ≥ 5 are not significant. In addition, the interquartile range (IQR) shows a high variation. Moreover, it is unclear in how many samples and in how many patients the percentage of CD34+ cells have been determined. From my point of view, Figure 2D could be removed. Along this line, the authors should explain, why an increased frequency of CD34+ hematopoietic progenitors in peripheral blood suggests that monocytes are mobilized from the bone marrow during acute HFRS (see Discussion, line 304-306).

**Author’s response to point 1:**

We thank reviewer 1 for their valuable comments on our manuscript. We agree with reviewer 1’s comment that the CD34+ hematopoietic progenitor data is not central to the conclusions made in the study. We found no significant differences in the frequencies of CD34+ cells between HFRS patients with a-SOFA scores <5 and ≥5 (former Figure 2D). For the interest of the reviewer 1, we have presented the frequencies of CD34+ cells in blood by individual patients to more clearly illustrate how many samples and patients were analyzed (Reviewer figure 1). Compared to uninfected controls (UCs, dotted line), increased frequencies of CD34+ cells were seen in 4 of 6 patients with a-SOFA scores ≥5 (Reviewer figure 1). In the revised version of the manuscript the CD34 progenitor data (former Figure 2D) has been moved to supplementary data (new Supplementary figure 1) and we have further clarified in the figure legend how many samples and patients were analyzed. As a consequence, we merged former figures 2A and B with Figure 1, and relabeled them as new Figures 1F and G. All successive figures are also renumbered in the text and discussion.

**Reviewer figure 1**: Frequency of CD34+ cells of total live cells in (A) all HFRS patients (n=16), (B) HFRS patients with a-SOFA scores <5 (n=10) and (C) HFRS patients with s-SOFA scores ≥5 (n=6). Dotted lines indicate median values from uninfected controls (n=3).

With our earlier (*Scholz et al, PLoS Pathog, 2017*) and current observations on altered frequencies of myeloid cells in blood during acute HFRS, we were interested in whether the kinetics of these changes could partly be explained by changes in the progenitor pool. CD34+ cells represent progenitors of all hematopoietic cells, including monocytes. Increased numbers of CD34+ progenitors have been reported in circulation following emergency myelopoiesis in response to infections and inflammatory challenges (*Kelly et al, Shock, 2020; Schulze et al, Immunity, 2019; Norris and Ernst, PLoS Pathog, 2018; Manz and Boettcher, Nat Rev Imm, 2014*). Our observation of a trend of increased CD34+ frequencies in circulation of HFRS patients may suggest ongoing myelopoiesis. In our revised version of the manuscript, potential myelopoiesis during early stages of HFRS is also supported by our new data of increased levels of macrophage colony stimulating factor (M-CSF) in plasma of HFRS patients, with acute infection as compared to convalescence (new Fig. 6H). Myelopoiesis in the bone marrow may lead to subsequent replenishment of the monocyte compartment in circulation, and differentiation of classical into nonclassical monocytes- the gap created by monocyte mobilization into the kidneys (supported by IHC data) and lungs (*Scholz et al, PLoS Pathog, 2017*). However, the discrete findings from our analyses are still only potential pieces to the complex puzzle of monocyte involvement in HFRS and this merits further investigation.

Subsequently, we have revised the manuscript results section at lines 147-148:

“Interestingly, this coincided with elevated CD34+ progenitor frequencies in blood during acute severe HFRS compared to during convalescence (Supplementary figures 1A-C).”

We have updated the text in the discussion at lines 340-346:

”As suggested by increased frequencies of CMs, CD34+ hematopoietic progenitors as well as elevated levels of CCL2 and M-CSF, it is likely that emergency myelopoiesis occurs in the bone marrow of patients with acute HFRS to replenish the early loss in monocytes and overall loss of NCMs. This may result in increased frequencies of circulating monocyte precursors later during acute disease and subsequently, normalization of monocyte frequencies to levels seen in UCs during the convalescent phase of HFRS, and merits further investigation in future studies.”

We have also changed the labeling of the previous Supplementary figure 1 to 3 (lines 160 and 700) and further clarified in the figure legend how many samples and patients were analyzed.

Due to merging of former figures 2A and B with Figure 1, and relabeling them as new Figures 1F and G, we have edited the manuscript text as follows:

Lines 141:

“Patients with more severe HFRS display delayed peak monocyte frequencies”

Line 143:

“…Figure 1F”

Line 146:

“…Figure 1G”

Lines 738-739:

“… thrombocytopenia**,** acute kidney injury and an overall increase in monocyte frequencies”

Lines 747-756:

“(F) Graph shows the total leukocyte counts (median ± interquartile range, IQR) during PUUV infection for patients stratified by severity as lower, i.e. a-SOFA score <5 (empty circles) or higher, i.e. a-SOFA score ≥5 (filled circles). (G) Graph shows median ± IQR monocyte frequencies to total live cells (classical + intermediate + nonclassical monocytes) during PUUV infection for patients stratified by severity as lower, i.e. a-SOFA score <5 (empty circles) or higher, i.e. a-SOFA score ≥5 (filled circles). Dotted line represents median values from UCs. Statistical differences between day 360 and other time points (B-E), and patients with lower and higher a-SOFA scores (F-G) were assessed using a generalized estimated equation (GEE) model in SPSS and differences were considered significant at p<0.05 (**p<0.01, ***p<0.001 and ****p<0.0001).

**Point 2:**

Figure 4 (showing kidney biopsies) and Figure 5 (showing cytofluorimetric analyses) are mixed up. According to the figure legends, Figure 4 should be labelled Figure 5 and vice versa.

**Author’s response to point 2:**

Reviewer 1 is absolutely correct, and we apologize for the error and the confusion. In the revised version of the manuscript, we have corrected the errors in the figure legends and in running text. Former Figure 4 displaying data from kidney biopsies is the new Figure 3, and former Figure 5 displaying flow cytometric data is the new Figure 4.

We have moved the corresponding text from the Results section and in the legend, to refer to the figures in the correct order as follows:

New Figure 3: lines 167- 181

New Figure 4: lines 183-197

Legend for Figure 3: lines 773-780

Legend for Figure 4: lines 782-795

Lines 171, 173:

“Figures 3A-D”

Line 171:

“Figures 3E-H”

Lines 192, 232, 769-770, 793:

For the sake of consistency across the manuscript, we have also substituted “mild” and “severe” with “… lower a-SOFA scores” and “… higher a-SOFA scores”, respectively.

Line 191:

We have also removed a sentence from the manuscript referring to maturation status of dendritic cell subsets in patients with HFRS (data not shown), to comply with journal requirements. The manuscript primarily focuses on monocytes, and we believe removing this statement does not affect our findings in a significant manner.

“~~No significant changes of HLA-DR, CCR2, CD62L or CCR7 were observed in DCs during the acute stage of HFRS (data not shown).~~”

**Point 3:**

In Figure 5 (labelled Figure 4) it is important to know the kidney diseases of the controls and other clinical data of the controls especially age distribution

**Author’s response to point 3:**

We agree with reviewer 1 that clinical data from both HFRS patients and non-HFRS patient controls that were biopsied to study kidney tissue is important. Due to the archival nature of these samples (collected between 1978-1989), there is a limit to the clinical information we have been able to obtain. We have summarized all clinical data available on the patients that provided kidney biopsies in revised Supplementary table 2 including age, gender, kidney disease diagnosis (when available), the days post symptom onset for each sample obtained, and their creatinine levels (max creatinine value for HFRS patients; creatinine level on the day of sampling for the control kidney samples). For a number of patients (n=6 of 43 for non-HFRS controls and n=5 of 37 HFRS patients), no specific kidney diagnosis was established/reported, even if all patients underwent kidney biopsy based on suspicion of kidney pathology. We have also added the kidney clinical condition of the samples displayed in revised Figure 3: Acute Tubulointerstitial Nephritis and to the figure legend. For a better comparison, we replaced the former control figures with IHC images from a non-HFRS patient also diagnosed with acute tubulointerstitial nephritis.

Lines 775-776:

“…both patients diagnosed with acute tubulointerstitial nephritis”

And finally, we have corrected the number of non-HFRS controls
Lines 169, 778:

43

**Point 4:**

The authors claim that monocytes are found in increased numbers in the kidneys of patients with acute HFRS. It is unclear, however, which cell type is really observed in the kidney biopsies from HFRS patients. Under inflammatory conditions, monocytes that extravasate into peripheral tissues develop into another cell type with morphological and functional features of dendritic cells (DC) or macrophages. For this reason, the HLA-DR+ and CD14+ or CD16+ cells detected in the kidney biopsies are not monocytes but monocyte-derived inflammatory DCs or macrophages. The authors should analyse additional markers to clarify this important point.

**Author’s response to point 4:**

Indeed, monocytes and DCs that extravasate into tissues during inflammatory conditions can differentiate to monocyte-derived inflammatory DCs or macrophages. To establish the identity and phenotype of the cells infiltrating the kidneys during HFRS, a preferred method, instead of immunohistochemistry (IHC), would be to perform multi-parametric flowcytometric analysis (similar to the analysis we performed on circulating cells) from patients. Preferably with matched blood samples taken at the same time point. However, the current availability of renal tissue and kidney biopsies (performed routinely a few decades ago but not any longer) from HFRS patients is very limited. Still, as reviewer 1 points out, it is important to clarify, to the extent possible, if the HLA-DR, CD14 and CD16 expressing cells seen to accumulate in the kidneys of HFRS patients exhibit macrophage-like phenotype. To this end, with the remaining biopsy material available, we performed additional IHC stainings for the macrophage marker CD68. We found a significant increase of the frequency of CD68+ cells in kidneys from HFRS patients compared to non-HFRS controls suggesting that at least a proportion of infiltrating monocytes differentiate to macrophages. However, to really pinpoint monocyte differentiation in tissues complementary techniques are needed along with available tissue samples. The CD68 IHC data is now included in the new Figures 3D and H. Figure 3D shows representative stainings for CD68 from a patient with acute HFRS and a control non-HFRS patient (both diagnosed with acute tubulointerstitial nephritis). Figure 3H shows quantification of %CD68+ cells in kidneys from both groups.

In line with the revisions to the figures, we have also updated the text as follows:

Lines 36, 105, 169, 170, 175, 365, 507, 511, 775, 777:

“…and CD68+ cells”

Lines 172:

“…likely monocyte-derived macrophages”

Lines 177, 363:

“…and monocyte-derived cells”

Lines 364-365:

“…and the presence of CD68-expressing macrophage-like cells suggests in situ monocyte differentiation into macrophages.”

Lines 177-181:

“…may contribute to the renal pathology observed during HFRS. We also noted that while the majority of monocytes detected in the kidneys during HFRS were predominantly CD14+, the high frequencies of CD16+ could explain the loss of NCMs from circulation. In addition, sustained mobilization of CD14+ CMs to renal tissues during HFRS (as seen in the biopsies) may contribute to decreased differentiation of CMs to IMs to NCMs in circulation.

**Point 5:**

The extravasation of monocytes into kidney tissue implies that kidney tissue is infected by PUUV. Indeed, a previous study of PUUV-infected patients has shown that hantaviral N protein is detected in podocytes in the glomeruli and in tubular epithelial cells (Krautkrämer et al., 2011). However, in the present study the monocyte-derived inflammatory cells localized predominantly to the tubulointerstitial space. It would be important to determine whether hantaviral N protein can be detected and whether it is located in the proximity of infected areas. Moreover, the authors should discuss how the virus is transported from the lung to the kidney.

**Author’s response to point 5:**

We agree with reviewer 1 that localization of the hantaviral N protein within the kidneys relative to the location of the infiltrating monocytic cells is likely important in understanding the role of monocytes in renal pathogenesis of HFRS and identifying potential targets for therapeutic interventions. Unfortunately, in the biopsy material we used for this study, we were unable to assay for the presence of the N antigen (or PUUV RNA), despite repeated attempts. One challenge, faced by us and the field in general, is the availability of specific reagents for N protein detection (e.g. monoclonal Abs) compatible with the material at hand. This is especially true for the current patient biopsy material, which was prepared decades ago using a relatively uncommon fixative, Bouin, which affects protein antigenicity in a different manner compared to the more common formaldehyde-containing fixatives. Bouin fixation occurs under acidic conditions whereas formalin fixation is pH neutral, which can affect protein antigens differently (*Tanca et al, Electrophoresis, 2012; Gagnon et al, Appl Immunohistochem Mol Morphol, 2011*). However, the markers we used were to identify our cells of interest in the tissues remained detectable, allowing us to speculate on their role in contributing to local inflammation, and in mitigating it.

We also thank reviewer 1 for highlighting an important reference, *Krautkrämer et al* (*J Virol, 2011*), where the authors showed disruption of tight junctions of tubular and glomerular cells, and demonstrated the presence of the hantaviral N antigen in the tubular and glomerular apparatus. While similar studies on HFRS patient kidney material are few, additional insights can be obtained into hantavirus pathology by relying on animal models. One such example is the 2008 study by *Sironen et al* (*PLoS One, 2008*), where the authors (including two co-authors of this manuscript) described the pathology of experimental infection of macaques with the Puumala virus (Kazan strain). The disease manifestation in the macaque model was deemed comparable and physiologically relevant to hantavirus disease in humans. Viral N protein was detected in the kidneys, spleen and liver. Within the kidneys, inflammatory cell infiltrates were described in the tubulointerstitial spaces, and sequential sections showed presence of both PUUV RNA and N protein in tubular epithelial cells and vascular endothelial cells surrounding the tubulointerstitial space. Therefore, one may speculate that the hantaviral N protein (and analogously, the virus) may be present in close proximity of tubulointerstitial spaces also in infected humans. However, this requires further investigation preferable using prospectively obtained patient material.

Reviewer 1 also suggested discussing how the virus is transported from the lungs to the kidneys. In general, endothelial cells are the target cells for hantaviruses. Upon inhalation of virus and exposure of the lungs, hantavirus infection and HFRS are typically accompanied by viremia, which suggests that circulation is a likely route of systemic dissemination for the virus (*Evander et al, J Clin Microbiol, 2007; Korva et al, Clin Microbiol Infect, 2013; Pettersson et al, Clin Microbiol Infect, 2014*). Hantaviruses can be viably isolated from plasma and have been detected in many bodily fluids including urine. The highest amounts of the virus are found in the lungs and in the kidneys (depending on the strain of the virus), which are both highly vascularized organs. We have included a comment on this a in the revised version of the manuscript (and cited an additional reference):

Lines 62-64:

“Hantaviruses enter the body primarily via inhalation and infection is typically accompanied by viremia, which suggests that circulation is a likely route of systemic dissemination for the virus (3)”

Lines 547–549:

“3. Evander M, Eriksson I, Pettersson L, Juto P, Ahlm C, Olsson GE, et al. Puumala hantavirus viremia diagnosed by real-time reverse transcriptase PCR using samples from patients with hemorrhagic fever and renal syndrome. J Clin Microbiol. 2007;45(8):2491-7.”

**Point 6:**

In order to determine whether HFRS directly impacted on blood monocyte function the author cultured and/or stimulated blood monocytes from HFRS patients and uninfected controls in vitro for 3 hours. They observed downregulation of CD16 indicating that the cells are no longer monocytes but developed into inflammatory DCs or macrophages. It has been shown that monocytes stimulated with hantavirus differentiate into DC-like cells (Markotic et al., 2007; Raftery et al., 2020). Thus, the authors should determine in which cell type monocytes develop under the conditions used.

**Author’s response to point 6:**

We agree with reviewer 1 that monocytes indeed differentiate into monocyte-derived dendritic cells (DCs)/macrophages upon exposure to hantaviruses as previously described (*Markotic et al, Viral immunol, 2007 and Raftery et al, Front Cell Infect Microbiol, 2020*). However, phenotypic differentiation and identification of monocyte-derived cells by flow cytometry likely require longer than the 3 hours of in vitro stimulation used in the present study. The rationale for such short stimulation is our previous observation, confirmed by other labs, that longer stimulation alters the surface expression of especially CD16 on myeloid cells (*Auffray et al, Science, 2007; Cros et al, Immunity, 2010; Baharom et al, J Immunol, 2016*). In our in vitro experiments, we wanted to use the same surface marker panel used to identify the MNP subsets in the patient samples including CD16, and therefore we used a short stimulation period. However, we would be excited to study the differentiation pathway(s) of monocytes in response to Hantavirus exposure that future studies, both using patient samples and cells from healthy controls. We have therefore cited *Raftery et al* (*Front Cell Infect Microbiol, 2020*) and highlighted this drawback of our experimental setup in the discussion of the revised manuscript with additional text as follows:

Lines 327-331:

“The CD11c+ cells, largely CD14+ classical monocytes, stimulated in vitro with TLR7/8L for 3 hours, also displayed downregulation of CD16. The brief duration of stimulation restricted our ability to study the potential differentiation of these cells in greater detail as previously reported (44). To understand the differentiation capacity of the monocytes remaining in circulation, future studies including trajectory analysis may provide additional insight.”

Lines 655-657:

“44. Raftery MJ, Lalwani P, Lutteke N, Kobak L, Giese T, Ulrich RG, et al. Replication in the Mononuclear Phagocyte System (MPS) as a Determinant of Hantavirus Pathogenicity. Front Cell Infect Microbiol. 2020;10:281.”

Lines 204-205:

We have also removed the phrase (data not shown) to comply with journal requirements, as reference (29) refers to the same phenomenon.

“Still, even 3 hours of culture resulted in downregulation of CD16 ~~(~~(29)~~, data not shown)~~, which prevented gating on separate monocyte subsets.”

**Point 7:**

The rationale behind stimulating monocytes with agonists of TLR7/8 is unclear, as hantaviruses are not known to trigger TLR7/8. In contrast, TLR3 and RIG-I-like receptors have been shown to function as PRRs for hantavirus infection (Handke et al., 2009; Lee et al., 2011; Kell et al., 2020). Thus, the observed impairment of TLR7/8 signaling pathways in monocytes from HFRS patients (Figure 6C) could be irrelevant for resolution of hantavirus infection.

**Author’s response to point 7:**

Reviewer 1 raises another important question about the TLRs utilized by hantaviruses. We would like to describe our rationale behind using TLR7/8L for the stimulation of PBMCs from HFRS patients (and uninfected controls). We and others have previously demonstrated the ability of monocytes to respond potently to TLR7/8 stimulation (*Baharom et al, JI, 2016; Metcalf et al, JI, 2017*), and this experimental design would help us to contrast monocyte responses in patients vs controls. However, as reviewer 1 highlighted in their comment (and the references listed by them), hantaviruses primarily rely on TLR3 and RLRs. Therefore, to address the question of disparity between TLR3 and TLR7/8L stimulation, we performed an additional experiment with PBMCs from one HFRS patient (similar to the experiment from new Figure 5), with both TLR3 and TLR7/8 agonists (Poly I:C and 3M-019, respectively), as displayed in Reviewer figure 2. We observed a similar pattern of responses in PBMCs, between the TLR7/8L and poly I:C stimulation, suggesting that, at least in vitro, both TLR ligands are comparable in their capacity to activate myeloid cells from HFRS patients. Unfortunately, due to limited availability of PBMCs from the HFRS patients, we were only able to perform these experiments with samples from one patient. Therefore, we presented this data here as a reviewer figure, rather than including it in the revised manuscript. In future hantavirus studies we will opt to use poly I:C for in vitro stimulation experiments similar to the ones described in this manuscript, in addition to a TLR7/8 agonist.

**Reviewer figure 2**: Frequency of (A) IL6+ and (B) TNF+ cells of total myeloid (CD11c+) cells in a representative HFRS patient, in response to stimulation with TLR 7/8 or TLR 3 agonists, as compared to unstimulated cells.

**Point 8:**

In Figure 6A, the differences between IL-6 production of stimulated versus unstimulated myeloid cells do not seem to be significant and the interquartile range (IQR) shows a high variation. In addition, the difference between IL-6 production of UCs versus HFRS patients does not seem to be significant.

**Author’s response to point 8:**

We agree with reviewer 1, that, unlike for TNF, the differences in IL-6 production between stimulated versus unstimulated myeloid cells from HFRS patients are not significant (former Figure 5A, new Supplementary figure 4C). However, the reason we highlighted this data is to illustrate that in the absence of stimulation, myeloid cells from HFRS patients in the earliest phase of HFRS produced significantly more IL-6 than myeloid cells from UCs. This data suggests that myeloid cells may be actively producing IL-6 and contributing to inflammation during acute HFRS. We apologize for displaying the figure in a manner that made it difficult to recognize this point clearly. Therefore, we have edited the presentation of the IL-6 data in the revised version of the manuscript to better present IL-6 expression in the absence of stimulation (Supplementary figure 4C). To not emphasize too strongly on this set of data we have moved all the IL-6 ICS data into supplementary data (Supplementary figures 4C-D). We also edited the running text as follows:

Lines 206-212:

“In the earliest acute phase of HFRS, a small proportion of the CD11c+ cells produced IL-6 without TLR stimulation, which gradually disappeared over the course of disease (Supplementary figures 4B-C). Upon TLR7/8L stimulation, 2-5% of the CD11c+ myeloid cells expressed IL-6 in both UC and acute and convalescent patient samples but we did not observe significant differences in their response to TLR stimulation (Supplementary figure 4D). IL-6 production in the absence of TLR stimulation negatively correlated with thrombocytopenia, but only in patients with higher a-SOFA scores (Supplementary figure 4E)”

**Point 9:**

In a previous study of PUUV-infected patients in Northern Sweden, the authors reported diminished numbers of all monocyte subsets (CM, IM, and NCM) and DCs subsets in peripheral blood during the acute stage of HFRS (Scholz et al., 2017). This is in stark contrast to the present study, in which acutely PUUV-infected patients from Central Finland showed increased numbers of circulating CMs and IMs whereas the numbers of NCMs were decreased. The authors explain this striking difference with an overall higher severity of HFRS in Central Finland. This explanation is not convincing as the Finish patients with a-SOFA < 5 (less severe disease) also show the same pattern: increased numbers of circulating CMs and IMs and decreased numbers of NCMs.

**Author’s response to point 9:**

Reviewer 1 is correct in that in our previous study on a Swedish HFRS cohort, we reported diminished numbers of blood monocyte and DC subsets during acute HFRS (*Scholz et al, PLoS Pathog, 2017*). We were surprised to observe a different pattern with the current Finnish cohort, despite similar staining protocols and gating strategies to identify the different blood monocyte and DC subsets. There are several factors which we have considered that may potentially explain the disparity in our findings. We have discussed whether differences in sample acquisition and handling could have contributed to the differences we see. This is a possibility but would best be addressed in side-by-side comparisons from point of patient inclusion to data analysis of both Finish and Swedish HFRS patients, which we have not been able to do to date. Furthermore, the Puumala virus strains circulating in Finland and Sweden are different, which may account for the differences we observe in our findings. In addition to potentially resulting in differences in disease severity, it is possible that the immunological response is also qualitatively different depending on the causative virus strain. In the future, to clarify similar issues, a prospective study comparing HFRS patients from Sweden and Finland, sampled and analyzed similarly and concurrently might be of great value. And finally, we apologize if we have overemphasized the effect of disease severity on the different patterns we report in our two studies. The clinical characterization of the patient cohorts suggest that the disease may be more severe in the Finnish cohort, as compared to the Swedish cohort. This is why we attributed our findings to “disease severity”. However, we now also added the following to the discussion:

Lines 309-313:

“Although highly speculative, the apparent increase in monocyte and DC frequencies towards the Finnish PUUV strain as opposed to the one circulating in Northern Sweden could also be attributed to the different strains. In any case, further work is needed to clarify potential differences in HFRS disease manifestation between the two geographical locations.”

**Point 10:**

In Figure 7B-G, the differences between patients with a-SOFA score < 5 and patients with a-SOFA score ≥ 5 are not significant. Moreover, there is confusion regarding Figure 7G. In the figure legend, Figure 7G (IL-18 and CCL2; against thrombocyte counts (109/L) is mentioned but not shown in the Figure. In the text, Figure 7G is referred to as showing elevated plasma creatinine levels correlating with thrombocytopenia.

**Author’s response to point 10:**

We apologize for the confusion in former Figure 7, which was entirely due to our error. We have corrected new Figure 6G (previously labelled 7H), as well as the legend:

Line 8118-820:

“(**F-G**) Lines display bivariate linear regression analysis between log_10_ cytokine values of (**F**) TNFα, IL-6, IL-10 and CCL-2 against thrombocyte counts (10^9^/L). (**G**) Lines…”

In the text, Figures 6G and 6H are referred to as correlations between cytokine levels and thrombocytopenia (former Figure 7F); and cytokine levels and creatinine levels (former Figure 7G) which we have now clarified:

Lines 236-240:

“We observed a correlation between cytokine levels and thrombocytopenia (Figure 6F); and between cytokine levels and plasma creatinine levels (Figure 6G), both hallmarks of acute PUUV-associated HFRS suggesting that, although not supported by a-SOFA based patient stratification, cytokine production in patients may still contribute to HFRS disease.”

As reviewer 1 also points out, there are no significant differences between the data shown in new Figures 6 B-E, between HFRS patients with a-SOFA scores <5 and ≥5. In the text, we have revised the corresponding statements as follows:

Lines 230-232:

“Somewhat surprisingly, when split by a-SOFA severity score, there was no consistent pattern in the plasma concentrations from patients with higher vs. lower scores (Figures 6B-E).

**Point 11:**

The headline of the last paragraph “CD16+ but not CD16– monocytes upregulate CD62L in response to direct virus exposure in vitro” is contradictory to the statement later in the paragraph (line 232-233) claiming that “Both CD16+ and CD16– monocytes upregulated CD62L after direct exposure to PUUV…”.

**Author’s response to point 11:**

In the revised version of the manuscript, we have rephrased the heading of the last paragraph in the results section to read “**CD16+ monocytes show more pronounced CD62L upregulation in response to direct virus exposure in vitro”**. The headline now better reflects the finding that the upregulation of CD62L by CD16+ monocytes is more pronounced than that observed in CD16– monocytes. The correction to the headline is at line 244.

**Point 12:**

In Figure 8B, it is important to know how many monocytes are infected with PUUV after direct exposure as compared to monocytes exposed to virus-infected endothelial cells. It cannot be ruled out that monocytes exposed to virus-infected endothelial cells are less efficiently infected and, therefore, do not upregulate CD62L. Moreover, differential susceptibility to PUUV infection could explain the differences of surface expression between CD16- and CD16+ monocytes.

**Author’s response to point 12:**

We apologize for the confusion regarding the rationale behind our experiment. To clarify, the purpose of this experiment was to attempt to mimic the activation of circulating monocytes during HFRS, and not to assess activation as a consequence of infection. Monocytes in blood likely become activated by PUUV in one of two ways- either by coming into direct contact with the virus in circulation, or by coming into contact with infected endothelial cells (ECs). In either situation, the monocytes themselves may not necessarily be productively infected. In our previous study, we observed that classical monocytes and CD1c myeloid dendritic cells were susceptible to PUUV infection, albeit at a very low frequency (1-3% of all cells) and only after extended culture (60 h) (*Scholz et al, PLoS Pathog, 2017*). Since the in vitro experiments performed in this study did not exceed 24 hours of monocyte exposure to PUUV or PUUV-infected cells, we estimated the likelihood of monocyte infection to be low (as also reported by *Raftery et al, Front Cell Infect Microbiol, 2020*), and therefore did not analyze the frequency of infected monocytes. As reviewer 1 pointed out, upregulation of CD62L by monocytes may indeed be influenced by the viral load they were exposed to, and the duration of exposure. Within the parameters of our experiments however, we wanted to examine the upregulation of CD62L in response to virus exposure and not infection or viral replication. Additionally, the monocytes were only exposed directly to the virus for 1 h, whereas the exposure to the infected endothelial cells was prolonged (20 h). But we only observed upregulation of CD62L with direct exposure (brief exposure) and not with exposure to infected endothelial cells (prolonged exposure). Also, with this experimental setup, we were able to identify differences (and some similarities) in the behavior of CD16+ and CD16– monocytes. The monocyte subsets differed in their choice of “activating” stimulus as well as their physiological response to the stimulus- the endothelial adhesion, migration and maturation markers they upregulate. However, the differential behavior of CD16+ and CD16– monocytes when exposed to the same stimulus suggests there are intrinsic differences between them. Our results are quite preliminary in this regard. To expand upon the differences between the monocyte subsets and their responses, detailed functional analyses are required, which in our opinion remain beyond the scope of this study.

**Reviewer #2: (No Response)**

**Author’s response:**

We are happy to note that Reviewer 2 found no major issues in the present study.

**Reviewer #3:**

**Point 1:**

A demonstration of the levels of GMCSF and MCSF in the patient plasma would significantly strength the authors hypothesis that loss of CD16+ monocytes from the blood leads to BM mobilization of progenitors. These are readily available luminex assays and levels of one or both would be expected to be elevated in the acute phase, and likely also higher in the more severely affected patients.

**Author’s response to point 1:**

In response to reviewer 3’s suggestion, we performed ELISA assays to measure GM-CSF and M-CSF in the plasma samples from HFRS patients. This data is now presented in new Figures 6A and H. However, at the time we performed the latest ELISA assays, the UC plasma samples used for the previous assay were unavailable, therefore the M-CSF and GM-CSF data is only from patients. We observed a significant upregulation of M-CSF (but not GM-CSF) in the acute HFRS plasma samples as compared to convalescence. We think this finding supports the idea of active myelopoiesis during acute HFRS and that at least some of the CD34+ progenitors will contribute to the circulating monocyte pool during the disease course. We edited the manuscript text accordingly as follows:

Lines 225-226:

“we measured the levels of various cytokines and chemokines in”

Lines 227-228:

“..IFNα, IFNγ, M-CSF and G-MCSF; and..”

Lines 240-242:

“We also detected a significant increase in the levels of M-CSF in patients during acute HFRS as compared to convalescent phase (Figure 6H) supporting the idea of active myelopoiesis during acute HFRS.”

Lines 341-347:

“As suggested by increased frequencies of CMs, CD34+ hematopoietic progenitors as well as elevated levels of CCL2 and M-CSF, it is likely that emergency myelopoiesis occurs in the bone marrow of patients with acute HFRS to replenish the early loss in monocytes and overall loss of NCMs. This may result in increased frequencies of circulating monocyte precursors later during acute disease and subsequently, normalization of monocyte frequencies to levels seen in UCs during the convalescent phase of HFRS, and merits further investigation in future studies.”

Line 811:

“(…as assayed by Luminex and ELISAs)”

Line 815:
“and grey represents no samples assayed.”

Lines 823-826:

“(H) Graph shows median ± IQR plasma concentrations (pg/mL) of M-CSF over the course of HFRS in patients. Statistical differences between day 360 and other time points were assessed using a generalized estimated equation (GEE) model in SPSS and differences were considered significant at p<0.05 (*p<0.05 and **p<0.01).”

**Part III – Minor Issues: Editorial and Data Presentation Modifications**
Please use this section for editorial suggestions as well as relatively minor modifications of existing data that would enhance clarity.

**Reviewer #1:**

**Point 1:**

For sake of clarity, in the legends to Figure 6 and Figure 8 the authors should describe the shown experiments in more detail. For example, it is unclear how long the mononuclear cells were cultured/stimulated in vitro and the MOI used for infection is not mentioned.

**Author’s response to point 1:**

We thank reviewer 1 for their suggestion and are glad to provide additional details in the figure legends. We have made edits as follows:

Line 800:

“… TLR 7/8 stimulation for 3 hr.”

Lines 835-836:

“… monocytes exposed to PUUV (MOI = 1) directly for 1 h followed by 19 hr of incubation with fresh media (left panels) or PUUV-infected (MOI = 10, for 20 h) BECs…”

**Point 2:**

In Figure 4B, the authors show that CCR2 expression on CM is not affected during acute HFRS. In their previous paper (Scholz et al., 2017), however, the authors report that CCR2 was downregulated on classical monocytes (CM) after PUUV infection in vitro. The authors should comment on this difference.

**Author’s response to point 2:**

We thank reviewer 1 for highlighting an important observation- in new Figure 4B. In the previous study (*Scholz et al, PLoS Pathog, 2017*), we were only able to assess CCR2 expression on monocytes exposed to Puumala virus in vitro and not the CCR2 expression on monocytes in patient samples. In the present study, we therefore measured CCR2 expression on blood monocytes (and DCs) in patient samples from acute HFRS and convalescence. While we observed differences in CCR2 expression on IMs and NCMs, CCR2 expression on CMs did not vary significantly. There may be several potential explanations for this finding; the CMs remaining in circulation may not yet have been exposed to the virus, or exposed briefly (since viremia is rapidly decreasing during hospitalization, see Fig 1E). The CMs which have come into contact with the virus or with infected endothelial cells may have migrated out of circulation and/or differentiated into monocyte-derived DCs or macrophages. Chemokine receptors are quite sensitive to culture conditions and may be reflective of inherent differences between the experimental conditions of in vitro and ex vivo studies. Lastly, CCR2 expression on monocytes may also vary depending on the different viral strains causing HFRS in the two cohorts.

We have clarified this and commented on CCR2 expression on monocytes in vivo vs. in vitro in the revised version of the manuscript’s discussion:

Lines 333-339:

“In our previous study (33), we observed downregulation of CCR2 by monocytes exposed to PUUV in vitro, which is in contrast to our observations in HFRS patients. The CMs we observed in circulation may not have been exposed to the virus, whereas monocytes that did encounter the virus may have migrated to the kidneys, as suggested by the kidney biopsy data. The disparity in observations between ex vivo and in vitro studies may be aided by chemokine receptors being quite sensitive to culture conditions, and by different viral strains being investigated in the studies.”

**Reviewer #2:**

**Point 1:**
I have some comments about Figures:
1. There is a substitution between Figure 4 and Figure 5. Figure 5 was uploaded instead of Figure 4 and Figure 4 instead of Figure 5.
2. On Figure 7 is correct 7G instead of 7H.
3. Figure 8D is not mentioned in the text (at results section). **Author’s response to point 1:**

We thank reviewer 2 for their comments on the figures. We apologize for the errors with figure numbers and legends. These errors have now been corrected in the revised version of the manuscript. We have also correctly referred to new Figure 7D in the text where the data in the figure was referred to:

Lines 271-273:

“Both CD16+ and CD16– monocytes displayed increased adhesion to PUUV-infected or TNFα-treated BECs suggesting that PUUV infection elevates the adhesive properties of endothelial cells towards monocytes (Figure 7D).”

**Point 3:**

Line 337: It is known or suspected which host genetics factors have a role in the monocyte response to infected endothelial cells?

**Author’s response to point 2:**

We thank reviewer 2 for highlighting an important aspect of the pathogenesis of hantaviruses- the association between host genetic factors and increased disease severity. Human leukocyte antigen (HLA) gene polymorphisms can influence susceptibility to infectious diseases. Previous reports have found an increased risk of severe disease in individuals with certain HLA-B and HLA-DR haplotypes, following infection with old and new world hantaviruses in Europe, Asia and the Americas. Polymorphisms in other relevant genes like TNF, C4 (complement protein 4), HPA-3 (integrin alpha-2b), VE-CDH5 (VE-cadherin endothelial tight junction) etc. have also been associated with increased risk of severe diseases (*Mäkelä et al, J Infect Dis, 2002; Charbonnel et al, Viruses, 2014*). A recent study has also demonstrated the association between an endothelial cell nitric oxide synthase gene polymorphism (G894T), and severe acute kidney injury during Puumala virus-caused HFRS (*Koskela et al, PLoS One, 2015*). However, the effect of the aforementioned polymorphisms on monocyte interactions with infected endothelial cells have not been studied in great detail. We have now cited and discussed these studies in the discussion as follows:

Lines 636-644:

37.	Mäkelä S, Mustonen J, Ala-Houhala I, Hurme M, Partanen J, Vapalahti O, et al. Human Leukocyte Antigen–B8-DR3 Is a More Important Risk Factor for Severe Puumala Hantavirus Infection than the Tumor Necrosis Factor–α(−308) G:A Polymorphism. J Infect Dis. 2002;186(6):843-6.

38.	Charbonnel N, Pages M, Sironen T, Henttonen H, Vapalahti O, Mustonen J, et al. Immunogenetic factors affecting susceptibility of humans and rodents to hantaviruses and the clinical course of hantaviral disease in humans. Viruses. 2014;6(5):2214-41.

39.	Koskela S, Laine O, Makela S, Pessi T, Tuomisto S, Huhtala H, et al. Endothelial Nitric Oxide Synthase G894T Polymorphism Associates with Disease Severity in Puumala Hantavirus Infection. PLoS One. 2015;10(11):e0142872.

Lines 306-309:

“Certain HLA-B and HLA-DR haplotypes have been associated with more severe disease following infection with old and new world hantaviruses, and polymorphisms in the TNF, VE-cadherin, integrin and endothelial nitric oxide synthase genes have been associated with increased risk of severe disease in HFRS (37-39).”

Lines 379-381:

“Rather, other factors including host genetics may play a role in the propensity of monocytes to upregulate CCR7 in response to infected endothelial cells, although this is currently only our speculation.”

**Point 3:**
Line 403: I would include more detailed information about performance of Luminex assay.

**Author’s response to point 3:**

We have now expanded the methodology of the Luminex assay as suggested by reviewer 2, as well as the ELISA assays performed upon the suggestion of reviewer 3:

Lines 447-455:

“**Cytokine and chemokine assays**

Cytokines and chemokines in plasma (1:2 dilution) were measured using a custom-designed 12-plex Luminex assay (for TNFα, IL-6, IFNα, IFNγ, IL-8, IL-10, IL-18, CCL2, CCL3, CCL7, IL-1β and IL-12p70) kit (R&D Systems) and analyzed on the Bio-Plex 200 instrument (Bio-Rad). Exact values were interpolated from standard curves (5PL logistic regression) on built-in Bio-Plex Software (Bio-Rad). M-CSF and GM-CSF in plasma was measured by enzyme-linked immunosorbent assay (ELISA) from Sigma and R&D Systems, respectively, according to manufacturer’s instructions. Exact values were interpolated from standard curves (5PL logistic regression) on GraphPad Prism version 9.0 (GraphPad Software).”

**Reviewer #3:**

**Point 1:**
Line 48: "noticeable" burden- as compared to an unnoticeable burden?

**Author’s response:**

We thank reviewer 3 for their comment and have rephrased the sentence at to say “clear burden”.

Line 62:

“pose a clear burden to human health.”

**Point 2:**
Table 2: units for Creatinine are incorrect.

**Author’s response to point 2:**

We thank reviewer 3 for bringing this to our attention, and we have now corrected the unit for creatinine in Table 2 at line 129.

**Point 3:**

There is no description of the disease course for the patients whose renal biopsies are used. Acknowledging that these samples are from 40+ years ago, can some clinical data be obtained? Day post symptom onset that the samples were obtained? Cre levels? Demographics?

**Author’s response:**

We agree with reviewer 3 that clinical data from both HFRS patients and non-HFRS patient controls that were biopsied to study kidney tissue is important. Due to the archival nature of these samples (collected 1978-1989), there is a limit to what clinical information we have been able to obtain. We have summarized all clinical data available on the patients that provided kidney biopsies in Supplementary table 2 including age, gender, kidney disease diagnosis (when available), the days post symptom onset for each sample obtained, and their creatinine levels (max creatinine value for HFRS patients; creatinine level on the day of sampling for the control kidney samples). For a number of patients (n=6 of 43 for non-HFRS controls and n=5 of 37 HFRS patients), no specific kidney diagnosis was established/reported, even if all patients underwent kidney biopsy based on suspicion of kidney pathology. We have also corrected the number of non-HFRS controls to 43 (lines 169, 778 and in Supplementary table 2), and the total number of biopsy samples to 80 (line 410).

**Point 4:**

T cell staining in the renal biopsies is also an important addition, since activated T cells could also be HLADR+ and the authors prior data demonstrated increased T cells in the lungs of HFRS patients. **Author’s response:**

Due to the limited availability of the biopsy material, we were only able to perform one additional set of stainings. As our study focuses on mononuclear phagocytes and not on adaptive immune responses, we prioritized identification of macrophage-like cells were present in the kidneys, and therefore, we stained the available sections with CD68. However, we do acknowledge the reviewer’s point that infiltrating lymphocytes can also contribute to the observed number of HLA-DR+ cells. In fact, in another study using the same biopsy material, we have observed increased numbers of B cells or plasma cells in the kidney, potentially contributing to the observed HLA-DR+ cell numbers (*Hepojoki et al., Manuscript in preparation*).

**Point 5:**

Authors should be careful to not attribute monocyte infiltration into the kidney to the pathology. Only correlation is demonstrated in this study. There are multiple instances of this throughout the manuscript.

**Author’s response to point 5:**

We agree with reviewer 3 that indeed, our data only demonstrated correlation. Therefore, we have rephrased our interpretation at the following instances in the manuscript:

Line 177:

“…during HFRS may contribute to the renal pathology observed during HFRS.”

Line 288:

“…and may contribute to the renal pathology during acute HFRS.”

Line 315

“…may suggest that HFRS is more severe…”

Line 358:

“infiltration and possibly, subsequent immune-mediated damage.”

**Point 6:**

Line 225: title to the section does not represent the data presented, since CD16- monocytes do up-regulate CD62L, just to a lesser extent. From the paragraph: "Both CD16+ and CD16– monocytes upregulated CD62L after direct exposure to PUUV or TLR7/8L, but in both cases CD16+ monocytes displayed higher level of CD62L induction than CD16– monocytes (Figure 8B, top panel)."

**Author’s response to point 6:**

In the revised version of the manuscript, we have rephrased the heading of the last paragraph in the results section to read “**CD16+ monocytes show more pronounced CD62L upregulation in response to direct virus exposure in vitro”**. The headline now better reflects the finding that the upregulation of CD62L by CD16+ monocytes is more pronounced than that observed in CD16– monocytes. The correction to the headline is at line 244.

**Point 7:**
Line 247: Be careful to not attribute a clinical patient finding based upon an in vitro expt.

**Author’s response to point 7:**

We have now rephrased the sentence to not overstate our findings and clarify that we are speculating about what happens in HFRS patients based on our in vitro data.

Line 267:

“…monocyte CCR7 upregulation in vitro, and perhaps in HFRS patients”

**Point 8:**

For flow: make sure representative gating strategy is shown for all analyses. There isn't one for CCR2/7 staining or for IL-6, TNFa in vitro expts. Also, need to make a table showing exact panel composition for each panel including the fluorphores used- can be supplementary.

**Author’s response to point 8:**

In the revised version of the manuscript, we have included complete gating strategies used in the analyses in this study (new Supplementary Figures 1, 2 and 4). The antibodies used for the flow stainings have been summarized in a new Supplementary Table 3. The text has been edited as follows:

Line 148:

“…Supplementary figures 1A-C)”

Line 155:

“…Supplementary figure 2A)”

Line 160:

“(Supplementary figure 3)”

Lines 432-433:

“(Panels summarized in Supplementary table 3)”

Lines 682-688:

“**Supplementary figure 1**. CD34+ progenitors are increased in acute HFRS patients (A) Gating strategy for identification of CD34+ progenitors from PBMCs by flow cytometry. (B) Flow plots depict CD34+ progenitors in a representative uninfected control and an HFRS patient over time (day 9-day 360).”

Lines 690-699:

**“Supplementary figure 2. Gating strategy used for identification of monocytes and dendritic cells from PBMCs.** (A) From total cells, live cells were identified as single cells negative for the live/dead marker. The lineage negative, HLA-DR+ cells were identified as cells expressing HLA-DR, but not CD3, CD19, CD20, CD56 or CD66. Myeloid cells were identified as CD11c-expressing cells and from the CD11c– population, CD123+ cells were identified as plasmacytoid dendritic cells (PDCs). From the CD11c+ cells, monocyte subsets were identified as CD14+CD16– classical monocytes (CM), CD14+CD16+ intermediate monocytes (IM) and CD14–CD16+ nonclassical monocytes (NCM). Myeloid DC subsets (CD1c+ and CD141+ MDCs) were identified from the CD14–CD16– cells. (B) Histograms show the relative expression of HLA-DR, CCR2, CD62L and CCR7 in classical, intermediate and nonclassical monocytes of a single representative HFRS patient over time (day 8 to day 360).”

Lines 716-731:

“**Supplementary figure 4. Gating strategy used for identifying TNFα and IL-6 producing myeloid cells with or without TLR stimulation.** (A) Flow plots depict TNFα-producing CD11c+ myeloid cells (gated as shown in supplementary figure 1) in a representative uninfected control and an HFRS patient over time (day 8-day 360), in the absence (top) or presence of TLR 7/8L stimulation (bottom) in vitro for 3hr. (B) Flow plots depict IL-6-producing CD11c+ myeloid cells (gated as shown in supplementary figure 1) in a representative uninfected control (UC) and an HFRS patient over time (day 8-day 360), in the absence of TLR 7/8L stimulation (bottom) in vitro for 3hr, and the frequencies are summarized in (C).”

Line 735:

“**Supplementary table 3:** **Flow cytometry panels**”

**Point 9:**

Figure 6: Please show all data points for this figure rather than bar graphs so reader can appreciate the spread of the data.

**Author’s response to point 9:**

Upon the suggestion of reviewer 1, we moved the IL-6 data from former Figures 6A and B to new Supplementary figure 4. We have also now illustrated all the individual data points in both figures to illustrate the spread of the data. We have also edited the legend and the running text to reflect the same:

Lines 723-725:

“(**D**) Graph displays frequency of IL-6 producing cells in CD11c+ myeloid cells in PBMCs in UCs (n=3) and HFRS patients (n=15) in the absence (circle) or presence (square) of TLR 7/8L stimulation for 3 hr.”

Lines 798-800:

“(**A**) Graph displays median frequency ± IQR TNFα producing cells in CD11c+ myeloid cells in PBMCs in UCs (n=3) and HFRS patients (n=15) in the absence (circle) or presence (square) of TLR 7/8L stimulation for 3h.”

**Point 10:**

For in vitro expts different ratios of monocytes to endothelial cells were used depending upon the expt. How were these ratios determined? How do they compare with what would be expected in vivo?

**Author’s response:**

As reviewer 3 has pointed out, we did use different ratios of monocytes to endothelial cells (ECs) depending on the assay. For the assay where monocytes were exposed to virus-infected ECs, which were performed in 24-well plates, monocytes were added at a ratio of 2.5 to 1. We arrived at this number after testing different combinations for recovery of viable cells after 20 hr incubation. The ratio ensured that the monocytes had sufficient surface area to bind to ECs. For the imaging experiments, performed in 96-well plates, we chose a higher monocyte to EC ratio (5:1) to saturate the ECs surface with monocytes and wash away unattached cells. It is very difficult to estimate how our in vitro monocyte-EC co-culture conditions compared with the in vivo situation. In vivo, monocytes rushing through circulation need to be attracted and immobilized by infected endothelial cells. NCMs, which perform patrolling functions in the vasculature, may possess some as yet-undescribed surface markers/ligand which allow them to “grab on” to endothelial adhesion molecules. We believe CD62L may be one such ligand, as we observe increased expression of CD62L on monocytes (especially CD16+ monocytes) during acute HFRS.
